# Risk of Immune-Related Pancreatitis in Patients with Solid Tumors Treated with Immune Checkpoint Inhibitors: Systematic Assessment with Meta-Analysis

**DOI:** 10.1155/2018/1027323

**Published:** 2018-06-03

**Authors:** Qiang Su, Xiao-chen Zhang, Chen-guang Zhang, Yan-li Hou, Yu-xia Yao, Bang-wei Cao

**Affiliations:** ^1^Department of Oncology, Beijing Friendship Hospital, Capital Medical University, Beijing 100050, China; ^2^Department of Biomedical Engineering, School of Medicine, Tsinghua University, Beijing 100084, China; ^3^Department of Biochemistry and Molecular Biology, School of Basic Medical Sciences, Capital Medical University, Beijing, China; ^4^Department of Ophthalmology, Beijing Friendship Hospital, Capital Medical University, Beijing 100050, China; ^5^Department of Digestive Diseases, Beijing Luhe Hospital, Capital Medical University, Beijing, China

## Abstract

We performed a systematic review and meta-analysis to determine the risk of immune-related pancreatitis associated with the treatment by immune checkpoint inhibitors (ICIs) for solid tumors. Eligible studies were selected from multiple databases including phase II/III randomized controlled trials (RCTs) with ICIs in solid tumor patients. The data were analyzed with Stata version 12.0 software. After excluding ineligible studies, a total of 15 clinical trials were considered eligible for the meta-analysis, which included 9099 patients. Compared with chemotherapy or placebo, the risk ratio (RR) for all-grade lipase elevation after CTLA-4 inhibitor treatment was 1.05 (95% confidence interval (CI): 1.01–2.24, *p* = 0.047). However, the risk for pancreatitis after ICI treatment in any subgroup was not significantly higher than that after control therapy. In addition, compared with ipilimumab/nivolumab alone, the RR for all-grade and high-grade lipase elevation under combination treatment of nivolumab and ipilimumab was 6.43 (95% CI: 1.43–28.99, *p* = 0.015) and 6.44 (95% CI: 1.39–29.79, *p* = 0.017), respectively, and the RR for all-grade amylase elevation under combination treatment was 6.08 (95% CI: 1.51–24.44, *p* = 0.011). Our meta-analysis has demonstrated that both CTLA-4 inhibitors alone and combination treatment of nivolumab and ipilimumab could increase the risk of amylase or lipase elevation, but not significantly increase the risk of pancreatitis when compared with controls.

## 1. Introduction

Up to now, cancers have been treated with surgery, chemotherapy, radiotherapy, and targeted molecular therapy including EGFR-TKI (epidermal growth factor receptor-tyrosine kinase inhibitor) [1]. Recently, immunotherapies involving immune checkpoint inhibitors (ICIs) include cytotoxic T lymphocyte-associated protein 4 (CTLA4) and programmed cell death protein 1 and ligand 1 (PD-1 and PD-L1) monoclonal antibodies. ICIs have emerged as a new effective treatment of advanced solid tumors such as advanced melanoma (MM), nonsmall cell lung cancer (NSCLC), and urothelial carcinoma [2–4]. In 2011, ipilimumab, the first ICI, has received US Food and Drug Administration (FDA) approval for the use in advanced melanoma [3, 4]. Since 2011, there have been additional five ICIs approved for the treatment of various solid tumors [2]. Unfortunately, when used both alone and combined, ICIs have generated new kinds of toxicity profiles, specifically referred to as immune-related adverse events (irAEs), which included but were not limited to thyroid dysfunction, colitis, pneumonitis, dermatitis, and hepatitis [5]. A less commonly seen irAE was immune-related pancreatitis [6–16]. The diagnosis of acute pancreatitis (AP) could be supported by increases of serum amylase and lipase. Values of serum amylase or lipase in excess of three times the upper limit of normal were characteristic of AP [17]. This study is conducted to evaluate the risk of immune-associated pancreatitis in cancer patients treated with ICIs. To our knowledge, this study is the first meta-analysis to report on the risk of immune-related pancreatitis under ICI treatment. We believe this meta-analysis study will improve awareness of the incidence and characteristics of immune-related pancreatitis, which may lead to more appropriate utilization of immune checkpoint inhibitors in clinical practice.

## 2. Methods

Our systematic review and meta-analysis was conducted according to the guidelines of the Cochrane Handbook for Systematic Reviews of Interventions [18] and the PRISMA Statement [19].

### 2.1. Strategy of Literature Searching

Random controlled trials (RCTs) exploring CTLA-4, PD-1, and PD-L1 antibodies in solid tumors were searched on the following databases: Embase, PubMed, and ClinicalTrials.gov. RCTs conducted between January 1990 and July 2017 were included. The medical subject heading (MeSH) terms included for searching the relevant studies contained one term that refers to cancer (neoplasm, carcinoma, cancer, or tumor), one term indicating the ICIs (anti-CTLA-4, anti-PD-1, ipilimumab, tremelimumab, nivolumab, pembrolizumab, atezolizumab, durvalumab, or avelumab), and one term related to randomized controlled trials, connected by “and” (Supplementary Table 1).

### 2.2. Inclusion and Exclusion Criteria

Studies with the following information were included in our meta-analysis: (1) phase II/III RCTs with primary endpoints such as overall survival (OS), progression-free survival (PFS), or objective response rate (ORR); (2) histologically confirmed solid cancer such as lung cancer and melanoma (MM); (3) containing the information of ICIs and pancreatitis or amylase or lipase; and (4) sharing some similarity in experimental method across different studies.

However, studies were excluded if they were (1) reviews, duplicate reports, letters, unfinished studies, or conference reports; (2) papers in languages other than English; (3) studies where pancreatitis could not be confirmed due to insufficient data; (4) studies conducted with cell lines, animal models, or on nonsolid cancers; (5) studies whose experimental method was substantially different from other selected RCTs; and (6) RCTs in phase I.

### 2.3. Data Extraction

Two reviewers (Qiang Su and Yan-li Hou) independently searched all the relevant studies and read the titles, abstracts, and full texts of the identified studies. Cases of disagreement were resolved through discussion with the third reviewer (Chen-guang Zhang). The following information was extracted from the selected studies: year of publication, authors' family names, methods of trails, number of ICI treatment type, number of control treatment, number of pancreatitis, amylase, and lipase of all grades (grades 1–5) and high grade (grades 3–5).

### 2.4. Data Analysis

In our meta-analysis, risk of bias analysis was performed using Review Manager 5.3 software (Cochrane Collaboration 2014, Nordic Cochrane Center, Copenhagen, Denmark). Two independent reviewers (Qiang Su and Yan-li Hou) assessed the quality of the included studies according to the Cochrane risk of bias tool. Specifically, the following seven domains were assessed: selection bias (including both *random sequence generation* and *allocation concealment*), performance bias, detection bias, attrition bias, reporting bias, and other biases. The Stata version 12.0 statistical software (Stata Corporation, College Station, Texas, USA) was used for meta-analysis. Risk ratio (RR) was used to estimate pancreatitis of grades 1–5 and grades 3–5. RR > 1.0 indicates higher risk or higher incidence of pancreatitis, amylase, or lipase in patients treated with ICIs. In addition, the *I* ^2^ statistics was used to assess the heterogeneity among the RCTs. *I* ^2^ values of <30%, 30%–59%, 60%–75%, and >75% were classified as low, moderate, substantial, and considerable heterogeneity, respectively [20]. We used the random effects model (REM) [21] to calculate pooled RR and 95% confidence interval (CI). Sources of heterogeneity were explored using subgroup analyses (different ICIs). The Begg's and Egger's tests were used to analyze the publication bias across RCTs. All *p* values were 2 tailed, and a probability level < 0.05 was considered statistically significant.

### 2.5. Quality Assessment

Our analysis was performed by pair-wise comparisons of the ICI arms and the control arms. Among the studies (see Table 1), there were five three-arm trials [11, 13, 16, 17, 22]. During the statistical process, one arm in each three-arm RCT maybe was entered twice in our database. The number of patients in this arm, which was used twice, was divided by two to avoid an increased influence of the arm on the overall result. In addition, we paid attention to the heterogeneity among the RCTs by using subgroup analysis. REM was employed for our meta-analysis to test and verify the statistical results.

## 3. Results

### 3.1. Selection of Studies

We initially identified 3877 studies from our search of the databases mentioned above. Among those 3877 studies, 15 RCTs met our inclusion criteria (Supplementary Figure 1). All 15 trials compared the effectiveness of ICI therapies with control treatments in solid tumors, representing data from a total of 9099 patients (Table 1). Among the 15 studies, four studies [7–9, 23] involved CTLA-4 antibodies (ipilimumab: 2 cohorts, *n* = 864; tremelimumab: 2 cohorts, *n* = 705), seven [10–13, 17, 24, 25] involved PD-1 antibodies (nivolumab: 3 cohorts, *n* = 605; pembrolizumab: 4 cohorts, *n* = 1748), one [14] involved PD-L1 (atezolizumab: *n* = 609), and the other three [15, 16, 22] studies involved combination treatment of nivolumab and ipilimumab (3 cohorts; *n* = 522). Eight trials [7, 8, 10, 13, 15–17] involved patients with malignant melanoma (MM), four [11, 12, 14, 24] involved patients with nonsmall cell lung cancer (NSCLC), and the other three [9, 22, 23] involved patients with mesothelioma, prostate cancer, or small cell lung cancer (SCLC).

Cochrane risk of bias tool was used to measure the quality of the included studies. The results are shown in Supplementary Figure 2. All of the included studies described the detail of random sequence generation and blinding of outcome assessment. However, some of them described incomplete outcome data and allocation concealment. Some studies failed to mention blinding of participants and personnel and selective reporting. Other indices of bias lacked specific description in the included clinical studies.

### 3.2. Analysis of Pancreatitis Risk: Comparison between ICI Treatments and Controls

**Figure 1:**
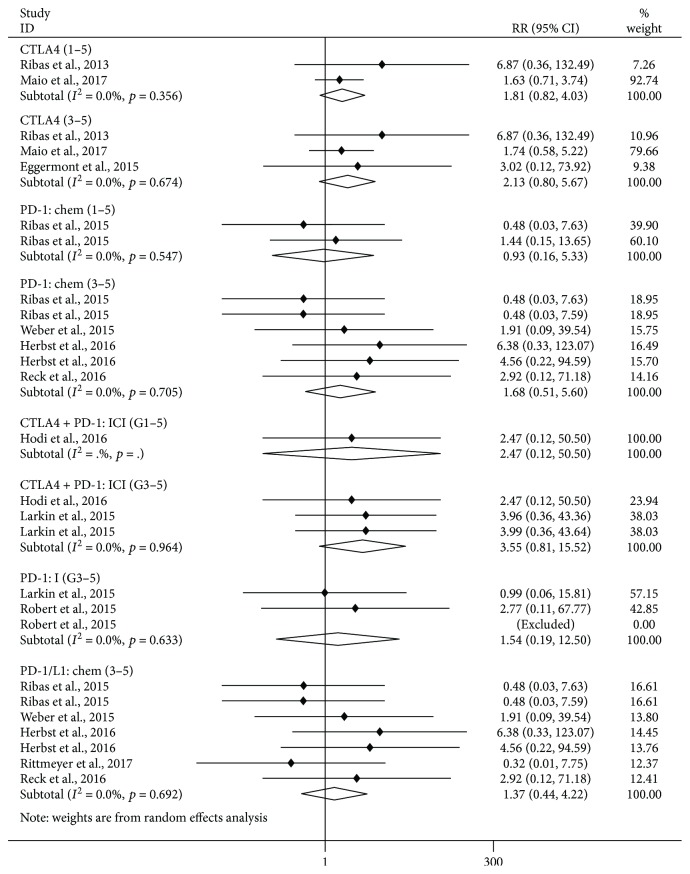
Forest plot analysis of pancreatitis in patients treated with PD-1/CTLA-4 antibodies compared with control therapy PD-1: chem: PD-1 inhibitor versus chemotherapy; PD-1: I: PD-1 inhibitor versus ipilimumab; PD-1/L1: chem: PD-1/L1 inhibitor versus chemotherapy; CTLA-4: CTLA-4 inhibitor versus chemotherapy/placebo; CTLA-4 + PD-1: ICI: nivolumab + ipilimumab subgroup versus nivolumab/ipilimumab; G1–5: grade1–5; G3–5: grade3–5.

As shown in Figure 1, compared with control treatments, there was no significant increase of risk of grade 1–5 pancreatitis in the CTLA-4 inhibitor subgroup (versus chemotherapy/placebo, RR 1.81, 95% CI: 0.82–4.03, *p* = 0.143) and in the PD-1 inhibitor subgroup (versus chemotherapy, RR = 0.93, 95% CI: 0.16–5.33, *p* = 0.937). Furthermore, we observed no significant increase in the risk of grade 3–5 pancreatitis in the CTLA-4 inhibitor subgroup (versus chemotherapy/placebo, RR = 2.13, 95% CI: 0.80–5.67, *p* = 0.130), in the PD-1 inhibitor subgroup (versus chemotherapy, RR = 1.68, 95% CI: 0.51–5.60, *p* = 0.395; versus ipilimumab, RR = 1.54, 95% CI: 0.19–12.50, *p* = 0.685), in the combination treatment of nivolumab and ipilimumab subgroup (versus ipilimumab, RR = 3.55, 95% CI: 0.81–15.52, *p* = 0.093), and in the PD-1/PD-L1 inhibitor subgroup (versus chemotherapy, RR 1.37, 95% CI: 0.44–4.22, *p* = 0.584).

### 3.3. Analysis of the Risk of Amylase Elevation: Comparison between ICI Treatments and Controls

**Figure 2:**
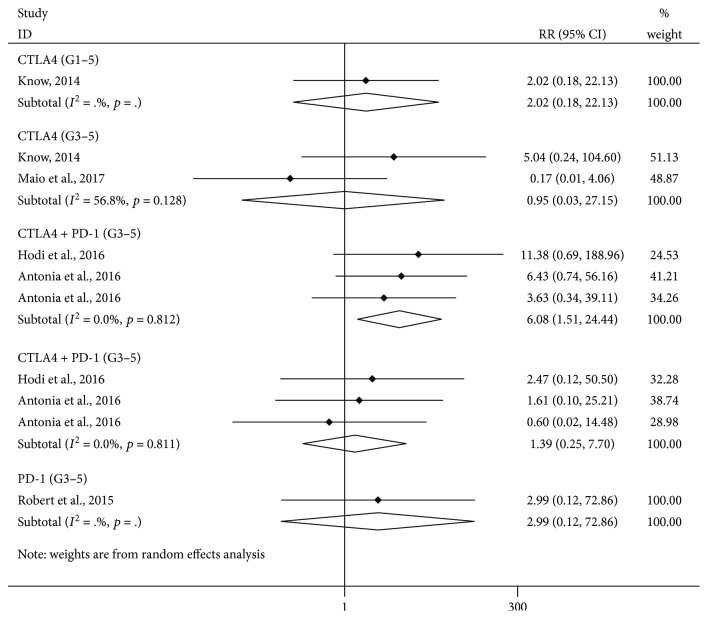
Forest plot analysis of amylase in patients treated with PD-1/CTLA-4 antibodies compared with control therapy CTLA-4: CTLA-4 inhibitor versus chemotherapy/placebo; PD-1: PD-1 inhibitor versus chemotherapy; CTLA-4 + PD-1: nivolumab + ipilimumab subgroup versus nivolumab/ipilimumab; G1–5: grade1–5; G3–5: grade3–5.

As shown in Figure 2, compared with control treatment, there was a significant increase of risk of grade 1–5 amylase elevation in the combination treatment of nivolumab and ipilimumab subgroup (versus ipilimumab or nivolumab alone, RR = 6.08, 95% CI: 1.51–24.44, *p* = 0.011). Furthermore, we observed no significant increase in the risk of grade 3–5 amylase elevation in the CTLA-4 inhibitor subgroup (versus chemotherapy/placebo, RR = 0.95, 95% CI: 0.03–27.15, *p* = 0.977) and in the combination treatment of nivolumab and ipilimumab subgroup (versus ipilimumab/nivolumab, RR = 1.39, 95% CI: 0.25–7.70, *p* = 0.708).

### 3.4. Analysis of the Risk of Lipase Elevation: Comparison between ICI Treatments and Controls

**Figure 3:**
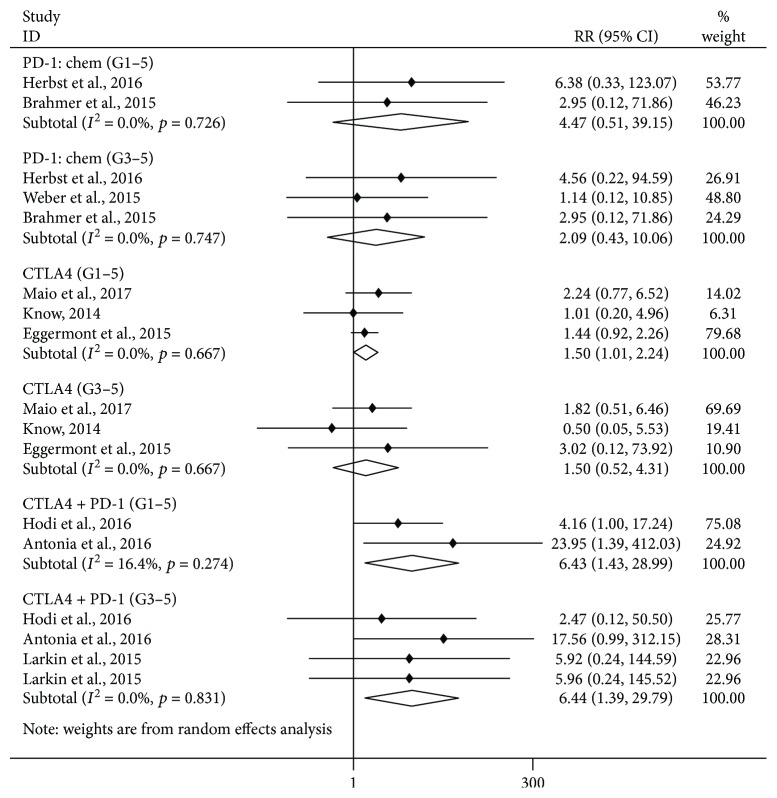
Forest plot analysis of lipase in patients treated with PD-1/CTLA-4 antibodies compared with control therapy. PD-1: chem: PD-1 inhibitor versus chemotherapy; CTLA-4: CTLA-4 inhibitor versus chemotherapy/placebo; CTLA-4 + PD1: nivolumab + ipilimumab subgroup versus nivolumab/ipilimumab; G1–5: grade1–5; G3–5: grade3–5.

As shown in Figure 3, we observed a significant increase in the risk of grade 1–5 lipase elevation in the CTLA-4 inhibitor subgroup (versus chemotherapy/placebo, RR = 1.50, 95% CI: 1.01–2.24, *p* = 0.047) and in the combination treatment of nivolumab and ipilimumab subgroup (versus ipilimumab/nivolumab, RR = 6.43, 95% CI: 1.43–28.99, *p* = 0.015). There was no significant increase in the risk of grade 1–5 lipase elevation in the PD-1 inhibitor subgroup (versus chemotherapy, RR = 4.47, 95% CI: 0.51–39.15, *p* = 0.176). Furthermore, we observed a significant increase in the risk of grade 3–5 lipase elevation in the combination treatment of nivolumab and ipilimumab subgroup (versus ipilimumab/nivolumab, RR = 6.44, 95% CI: 1.39–29.79, *p* = 0.017), but not in the CTLA-4 inhibitor subgroup (versus chemotherapy/placebo, RR = 1.50, 95% CI: 0.52–4.31, *p* = 0.451), or in the PD-1 inhibitor subgroup (versus chemotherapy, RR = 2.09, 95% CI: 0.43–10.06, *p* = 0.359).

#### 3.4.1. Heterogeneity of All the Subgroups

Heterogeneity in meta-analysis refers to the variation in study outcomes between studies. In nearly all the subgroups, we found the low overall heterogeneity of grade 1–5/3–5 pancreatitis, amylase, and lipase elevation incidence which displayed *I* ^2^ values of 0.0%, but not in this subgroup (CTLA-4 inhibitor subgroup versus chemotherapy/placebo: *I* ^2^ = 56.8%, *p* = 0.128).

### 3.5. Analysis of Publication Bias

Egger's test and Begg's test, conducted in Stata 12.0 software, were utilized to evaluate the publication bias between different RCTs. As presented in Supplementary Table 2 and Supplementary Figure 3, all *p* values were >0.05 after both tests. Therefore, there was no significant publication bias in this meta-analysis.

## 4. Discussion

ICI-induced pancreatitis remains a complicated irAE. The incidence of immune-related pancreatitis caused by ICIs is rare (CTLA4: 0.9–3%, PD-1: 0.5–1.6%, CTLA4 + PD-1: 1.2–2.1%) [6–16]. Since ICI-induced pancreatitis can be considered an immune-related pancreatitis, it is reasonable to propose that its diagnosis can be based on the diagnostic criteria of autoimmune pancreatitis (AIP) [26, 27]; the diagnosis of autoimmune pancreatitis is based on results from these five factors: imaging, serology, histology, extrapancreatic involvement, and perhaps steroid responsiveness. Although the mechanism of immune-induced pancreatitis caused by ICIs is largely unknown, it is postulated that ICIs could unavoidably disturb the balance of autologous tolerance, which could result in some immune-related side effects.

Our meta-analysis shows that compared with chemotherapy or placebo, using CTLA-4 inhibitors as single treatment significantly increases (RR = 1.50) the risk of all-grade lipase elevation among solid cancer patients. The risk of pancreatitis was not significantly elevated under CTLA-4 and PD-1 inhibitors alone or in combination. Compared with ipilimumab or nivolumab alone, combination treatment with nivolumab and ipilimumab might increase the risk of all-grade lipase elevation (RR = 6.43) and amylase elevation (RR = 1.39), as well as the risk of high-grade lipase elevation (RR = 6.44) among solid cancer patients.

We found that the CTLA-4 inhibitor increased the risk of lipase elevation (all-grade); however, neither CTLA-4 nor PD-1 inhibitor increased the risk of pancreatitis. Such a discrepancy between some elevated serologic test results and clinically significant pancreatitis further supported our proposal to use the diagnostic criteria of AIP to confirm ICI-induced pancreatitis. According to the mentioned criteria of ICI-induced pancreatitis, we noted that amylase/lipase elevation was only one out of the five key aspects for diagnosis; images including contrast-enhanced computed tomography (CT) or magnetic resonance imaging (MRI) seem more important clinically to discern immune-related pancreatitis [26]. Consistently, because of the rare incidence of ICI-induced pancreatitis [6–16, 28, 29], some studies propose that routine monitoring of amylase/lipase in asymptomatic individuals is not recommended. Also, the mechanism of elevated amylase/lipase in solid cancer patients treated with ICIs in the absence of clinically significant pancreatitis remains unclear. Given that combined ipilimumab and nivolumab results in a more frequent elevation of amylase and lipase suggests that the elevation of amylase and lipase is likely immune related.

Undoubtedly, the meta-analysis itself based upon published data had some limitations [30]. One limitation of this meta-analysis was the lack of individual patient data, the use of which would have provided more detail about immune-related pancreatitis with ICIs. Secondly, because of the paucity of the study number on immune-related pancreatitis, the sensitivity analysis was not employed in this meta-analysis. Thirdly, imaging data with CT or MRI to contain radiographic evidence of pancreatitis was absent. However, we have made efforts on the overall quality assessment that may make our results more steady and credible: (1) two independent reviewers searched all the relevant trails with well-defined inclusion criteria. They assessed the appropriate studies for meta-analysis evaluated by using PICO chart and assessed the risk of bias for the included RCTs according to the Cochrane Handbook. (2) Two independent reviewers verified data in our meta-analysis that was obtained from pair-wise comparisons. (3) The REM was statistically employed in this meta-analysis.

With the increasing morbidity of cancer and the lengthening of overall survival, although the incidence is very low, the cases of ICI-induced pancreatitis are estimated to grow [31, 32]. These should be useful in order for the clinicians to comprehend the incidence and risk of ICI-induced pancreatitis. Further study on the molecular mechanisms underlying ICI-induced pancreatitis could help us to prevent or relieve this adverse event during ICI treatment [33].

## 5. Conclusion

In summary, this meta-analysis study has demonstrated that CTLA-4 inhibitor therapy may result in a higher risk of all-grade lipase elevation when compared to chemotherapy. However, neither CTLA-4 nor PD-1 inhibitor when given alone or in combination increased the risk of immune-induced pancreatitis when compared to controls. Compared with nivolumab or ipilimumab, the combination of nivolumab and ipilimumab could increase the risk of all-grade and high-grade lipase elevation, as well as the risk of all-grade amylase elevation.

## Figures and Tables

**Table 1:** Characteristics of the eligible RCTs.

Study (year)	Study type	Histology	Endpoint	Treatment arm	Patient (no.)	Pancreatitis		AMY		Lipase	
						(G1–5)	(G3–5)	(1–5)	(3–5)	(1–5)	(3–5)
Ribas et al., 2013 [6]	RCT III	MM	OS	Tremelimumab at 15 mg/kg 90 ds	325	3	3	NA	NA	NA	NA
				Chemotherapy control	319	0	0	NA	NA	NA	NA

Know, 2014	RCT III	prostate Ca	OS	Ipilimumab 10 mg/kg q3w	393	NA	NA	2	2	3	1
				Placebo	396	NA	NA	1	0	3	2

Eggermont et al., 2015 [7]	RCT III	MM	PFS	Ipilimumab 10 mg/kg q3w	471	NA	1	NA	NA	43	1
				Placebo	474	NA	0	NA	NA	30	0

Maio et al., 2017 [8]	RCT IIb	Mesothelioma	OS	Tremelimumab 10 mg/kg q4w	380	2	1	NA	0	18	11
				Placebo	189	0	0	NA	1	4	3

Brahmer et al., 2015 [24]	RCT III	NSCLC	OS	Nivolumab 3 mg/kg q2w	131	NA	NA	NA	NA	1	1
				Chemotherapy control	129	NA	NA	NA	NA	0	0

Robert et al., 2011 [4]	RCT III	MM	OS	Nivolumab 3 mg/kg q2w	206	NA	NA	NA	1	NA	NA
				Chemotherapy control	205	NA	NA	NA	0	NA	NA

Weber et al., 2015 [9]	RCT III	MM	ORR	Nivolumab 3 mg/kg q2w	268	NA	2	NA	NA	NA	3
				Chemotherapy control	102	NA	0	NA	NA	NA	1

Herbst et al., 2016 [10]	RCT III	NSCLC	OS	Pembrolizumab 2 mg/kg q2w	339	3	2	NA	NA	NA	NA
				Pembrolizumab 10 mg/kg q2w	343	0	0	NA	NA	NA	NA
				Chemotherapy control	309	0	0	NA	NA	NA	NA

Reck et al., 2016 [11]	RCTIII	NSCLC	PFS	Pembrolizumab 200 mg q3w	154	NA	1	NA	NA	NA	NA
				Chemotherapy control	150	NA	0	NA	NA	NA	NA

Ribas et al., 2015 [12]	RCT II	MM	ORR	Pembrolizumab 2 mg/kg q2w	178	1	1	NA	NA	NA	NA
				Pembrolizumab 10 mg/kg q2w	179	3	1	NA	NA	NA	NA
				Chemotherapy control	171	1	1	NA	NA	NA	NA

Rittmeyer et al., 2017 [13]	RCT II	NSCLC	OS	Atezolizumab 1200 mg q3w	609	NA	0	NA	NA	NA	NA
				Chemotherapy control	578	NA	1	NA	NA	NA	NA

Hodi et al., 2016 [14]	RCT II	MM	ORR	Ipilimumab 3 mg/kg q3w +nivolumab 1 mg/kg q3w	94	2	2	11	2	17	2
				Ipilimumab 3 mg/kg q3w	46	0	0	0	0	2	0

Antonia et al., 2016 [22]	RCT II	SCLC	ORR	Ipilimumab 3 mg/kg q3w +nivolumab 1 mg/kg q3w	61	NA	NA	4	1	7	5
				Ipilimumab 1 mg/kg q3w +nivolumab 3 mg/kg q3w	54	NA	NA	2	0	0	0
				Nivolumab 3 mg/kg q2w	98	NA	NA	1	1	0	0

Larkin et al., 2015 [15]	RCT III	MM	OS/PFS	Ipilimumab 3 mg/kg q3w +nivolumab 1 mg/kg q3w	313	NA	2	NA	NA	NA	2
				Nivolumab 3 mg/kg q3w	313	NA	1	NA	NA	NA	0
				Ipilimumab 3 mg/kg q3w	311	NA	1	NA	NA	NA	0

Robert et al., 2015 [16]	RCT III	MM	OS	Pembrolizumab 10 mg/kg q2w	278	NA	0	NA	NA	NA	NA
				Pembrolizumab 10 mg/kg q3w	277	NA	1	NA	NA	NA	NA
				Ipilimumab 3 mg/kg q3w	256	NA	0	NA	NA	NA	NA

MM: melanoma; NSCLC: nonsmall cell lung cancer; DTIC: Dacarbazine; gp100: gp100 vaccine; DOX: docetaxel; OS: overall survival; ORR: objective response rate; NA: not available.

## Supplementary Materials

Supplementary Materials Supplementary file (including Supplementary Tables 1-2 and Supplementary Figures 1–3).
