# Supplementary material for: Risk of Immune-Related Pancreatitis in Patients with Solid Tumors Treated with Immune Checkpoint Inhibitors: Systematic Assessment with Meta-Analysis
Source: J Immunol Res. 2018 Jun 3;2018:1027323. doi: 10.1155/2018/1027323 (PMC6008648; doi:10.1155/2018/1027323)

Supplementary Table1: Search performed in Pubmed and Embase

<table>
<tr><td colspan="3">PubMed</td></tr>
<tr><td>#1</td><td>(((((((((Neoplasms) OR Neoplasia) OR Neoplasias) OR Neoplasm) OR Tumors) OR Tumor) OR Malignancy) OR Malignancies) OR Cancer) OR Cancers</td><td>4029451</td></tr>
<tr><td>#2</td><td>(((((((((nivolumab) OR MDX-1106) OR ONO-4538) OR BMS-936558) OR Opdivo))
OR
((((pembrolizumab) OR lambrolizumab) OR Keytruda) OR MK-3475)))
OR
(((Durvalumab)
OR
(((((((atezolizumab) OR anti-PDL1) OR immunoglobulin g1,anti (human cd antigens cd274) (human monoclonal mdpl3280a heavy chain) , disulfide with human monoclonal mdpl3280a kappa-chain, dimer) OR MPDL3280A) OR tecentriq) OR RG7446) OR RG-7446))
OR
((avelumab) OR MSB0010718C)))
OR
(((((((((((tremelimumab) OR ticilimumab) OR CP 675) OR CP675 cpd) OR CP-675) OR CP-675,206) OR CP-675206) OR CP675206) OR CP 675206))
OR
((((((((((Ipilimumab) OR Anti CTLA 4 MAb Ipilimumab) OR Anti-CTLA-4 MAb Ipilimumab) OR Ipilimumab, Anti-CTLA-4 MAb) OR Yervoy) OR MDX010) OR MDX 010) OR MDX-010 M) OR DX-CTLA-4) OR MDX CTLA 4</td><td>8497</td></tr>
<tr><td>#3</td><td>(((((((randomized controlled trail) OR controlled clinicaltrail) OR randomized) OR randomly) OR trial))</td><td>949142</td></tr>
<tr><td>#4</td><td>#1 AND #2 AND #3</td><td>Final search
1262</td></tr>
<tr><td colspan="3">EMBASE</td></tr>
<tr><td>#1</td><td>acral AND ('tumor'/exp OR tumor) OR 'neoplasms'/exp OR neoplasms OR (acral AND ('tumour'/exp OR tumour)) OR (('neoplasms'/exp OR neoplasms) AND by AND histologic AND type) OR (neoplasms, AND cystic, AND mucinous, AND serous) OR (neoplasms, AND embryonal AND mixed) OR (neoplasms, AND ('germ'/exp OR germ) AND ('cell'/exp OR cell) AND embryonal;) OR (neoplasms, AND glandular AND epithelial;) OR (neoplasms, AND 'hormone dependent;') OR (neoplasms, AND 'post traumatic') OR (neoplastic AND ('disease'/exp OR disease)) OR 'tumor'/exp OR tumor OR 'tumour'/exp OR tumour</td><td>4766232</td></tr>
<tr><td>#2</td><td>'ipilimumab'/exp OR ipilimumab OR (bms AND 734016;) OR bms734016; OR (mdx AND 010) OR (mdx AND 101;) OR 'mdx010'/exp OR mdx 010 OR 'mdx101'/exp OR mdx101 OR strentarga; OR 'yervoy'/exp OR yervoy
OR
'ticilimumab'/exp OR ticilimumab OR (cp AND 675, AND 206) OR (cp AND 675206) OR (cp AND 675 AND 206) OR (cp675, AND 206) OR 'cp675206'/exp OR cp675206 OR 'tremelimumab'/exp OR tremelimumab
OR
'nivolumab'/exp OR nivolumab OR (bms AND 936558) OR 'bms936558'/exp OR bms936558 OR (mdx AND 1106) OR (ono AND 4538) OR 'mdx1106'/exp OR mdx1106 OR 'ono4538'/exp OR ono4538 OR 'opdivo'/exp OR opdivo
OR
'pembrolizumab'/exp OR pembrolizumab OR 'keytruda'/exp OR keytruda OR 'lambrolizumab'/exp OR lambrolizumab OR (mk AND 3475) OR 'mk3475'/exp OR mk3475
OR
'avelumab'/exp OR avelumab OR bavenci OR (msb AND 0010718) OR (msb AND 10682) OR (msb AND 0010682) OR msb0010718c; OR msb</td><td>14116</td></tr>
</table>

	10682; OR 'msb10718c'/exp OR msb10718c<br>OR<br>'atezolizumab'/exp OR atezolizumab OR (monoclonal AND ('antibody'/exp OR antibody) AND mpdl AND 3280a) OR (monoclonal AND ('antibody'/exp OR antibody) AND ('mpdl3280a'/exp OR mpdl3280a)) OR (mpdl AND 3280a) OR 'mpdl3280a'/exp OR mpdl3280a OR (rg AND 7446) OR 'rg7446'/exp OR rg7446 OR 'tecentriq'/exp OR tecentriq OR 'tecntriq'/exp OR tecntriq<br>OR<br>'durvalumab'/exp OR durvalumab OR (medi AND 4736) OR 'medi4736'/exp OR medi4736	
#3	randomized AND controlled AND ('trial'/exp OR trial) OR (controlled AND trial, AND randomized;) OR 'randomized controlled trial'/exp OR 'randomized controlled trial' OR (pragmatic AND ('clinical'/exp OR clinical) AND trials) OR (randomised AND controlled AND ('study'/exp OR study)) OR (randomised AND controlled AND ('trial'/exp OR trial)) OR (randomized AND controlled AND study;) OR (trial, AND randomized AND controlled)	***755145***
#4	***#1 AND #2 AND #3***	***Final search***<br>***2615***

Supplementary Figure1: Flowchart depicting the RCTs selection process

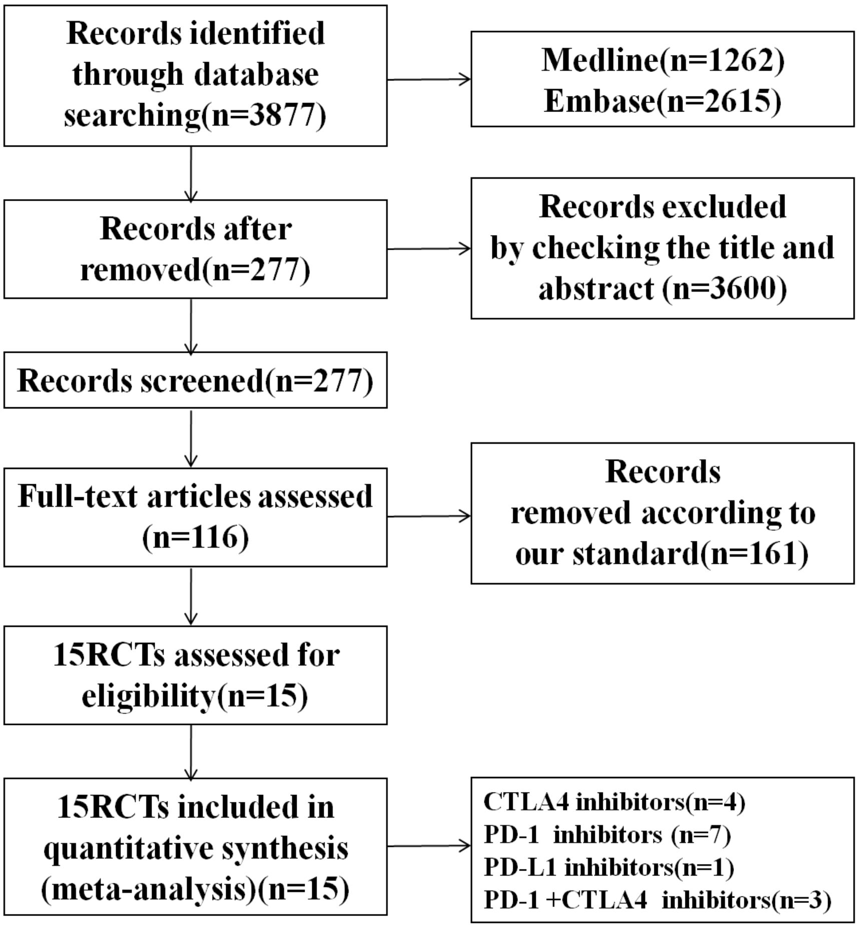

Supplementary Figure2: Risk of bias summary.

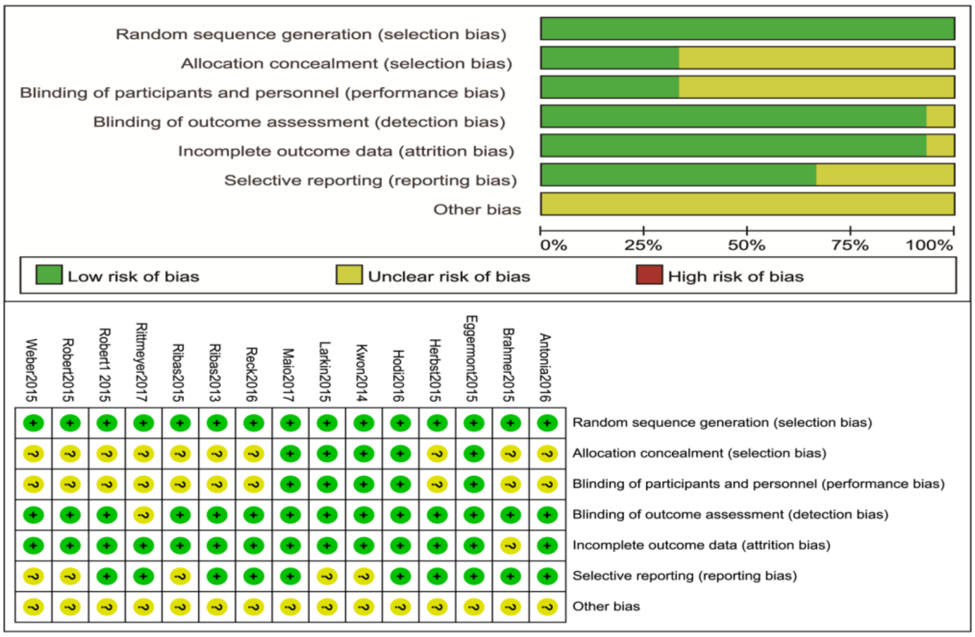

(A): Bar chart comparing percentage risk of bias for each included RCT. Low risk of bias (light black), high risk of bias (middle black), and unclear risk of bias (dark black). **(B):** Risk of bias for each included RCT, representing low risk of bias (+), high risk of bias (-), and unclear risk of bias (?).

Supplementary Table2: Evaluation of publication bias with Begg's test and Egger's test

	trails	Heterogeneity		RR（95%CI）	Begg's test		Egger's test	
		**P**	**I^2**		**Z**	**P**	**T**	**P**
**PD-1:pancretitis (3-5)**	6	0.705	0.0%	1.68(0.51,5.60)	0.75	0.45	1.33	0.25
**PD-1/L1:pancretitis (3-5)**	7	0.692	0.0%	1.37(0.44,4.22)	0.30	0.76	0.62	0.56
**CTLA4:lipase (1-5)**	3	0.667	0.0%	1.50(1.01,2.24)	0.00	1.00	0.25	0.85
**CTLA4:lipase (3-5)**	3	0.585	0.0%	1.50(0.52,4.31)	0.00	1.00	0.03	0.98
**Nov+Ipi:lipase (3-5)**	4	0.831	0.0%	6.44(1.39,29.79)	-0.34	1.00	-1.36	0.31

PD-1: PD-1 inhibitor subgroup; PD-1/L1:PD-1/L1 inhibitor subgroup;CTLA-4:CTLA-4 inhibitor subgroup; 1-5:grade1-5; G3-5:grade3-5.

Supplementary Figure3: Begg's funnel plot

(1.1) Begg's funnel plot for pancretitis (Grade3-5) in patients treated with *PD-1 inhibitors* versus chemotherapy

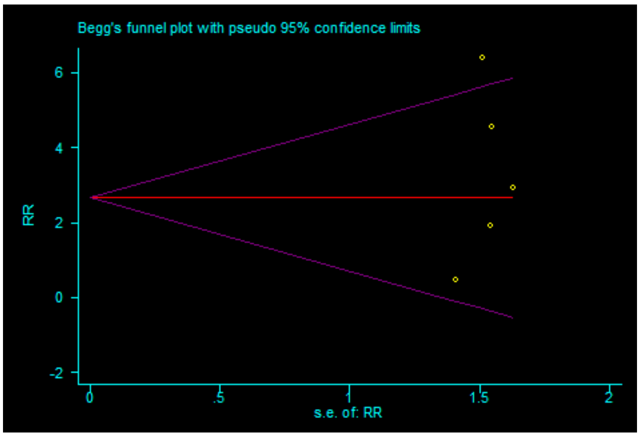

(1.2) Begg's funnel plot for pancretitis (Grade3-5) in patients treated with *PD-1/PD-L1 inhibitors* versus chemotherapy

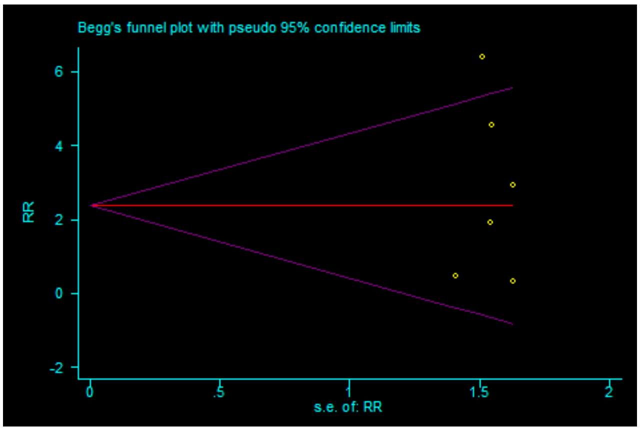

(2.1) Begg's funnel plot for lipase (Grade1-5) in patients treated with *CTLA4 inhibitors* versus chemotherapy/placebo

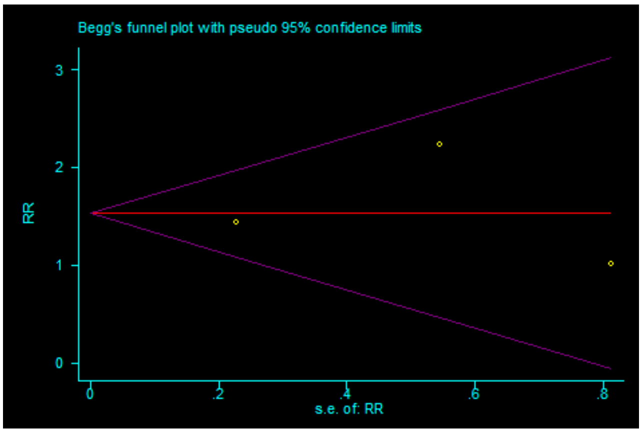

(2.2) Begg's funnel plot for lipase (Grade3-5) in patients treated with *CTLA4 inhibitors* versus chemotherapy/placebo

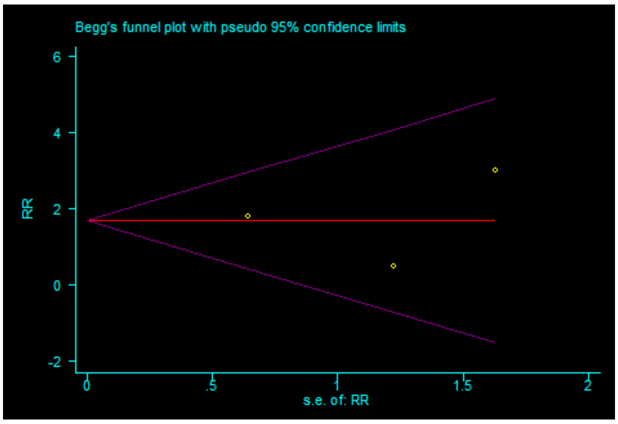

(2.3) Begg's funnel plot for lipase (Grade3-5) in patients treated with *nivolumab+ipilimumab* subgroup versus nivolumab/ipilimumab

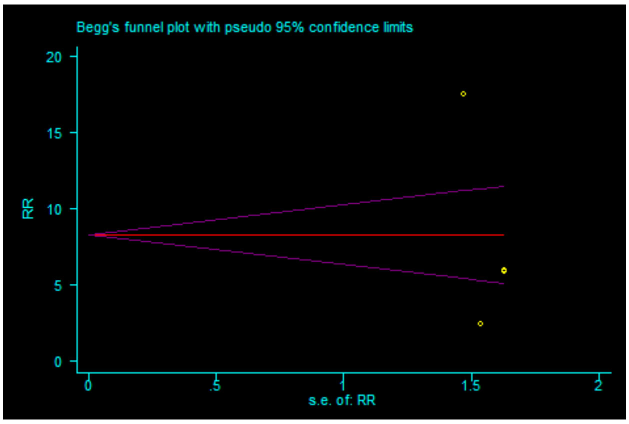

Supplement: Supplementary Materials — Supplementary file (including Supplementary Tables 1-2 and Supplementary Figures 1–3). [file 1027323.f1.pdf]
